# Pan-Cancer Analysis Reveals Long Non-coding RNA (lncRNA) Embryonic Stem Cell-Related Gene (ESRG) as a Promising Diagnostic and Prognostic Biomarker

**DOI:** 10.7759/cureus.67389

**Published:** 2024-08-21

**Authors:** Samira M Fageer, Marwa F Alamin, Areig M Attaelmanan, Mohamed Alfaki

**Affiliations:** 1 Microbiology and Molecular Biology, Bioscience Research Institute, Khartoum, SDN; 2 Molecular Biology, Institute of Endemic Disease, Khartoum University, Khartoum, SDN; 3 Biotechnology, Newcastle University, Newcastle Upon Tyne, GBR; 4 Research, Sidra Medicine, Doha, QAT

**Keywords:** lgg, bioinformatics analysis, immune infiltration, diagnostic biomarker, prognostic biomarker, lusc, read, coad, hesrg, esrg

## Abstract

Background: Embryonic stem cell-related gene (ESRG; also known as HESRG) is a long non-coding RNA (lncRNA). It is involved in the regulation of human pluripotent stem cells (hPSCs) self-renewal. ESRG gene has the ability to interact with chromatins, different RNA types, and RNA binding proteins (RBP); thus making ESRG be considered an oncogenic lncRNA, where its expression is detected in various tumor tissues. This study aimed to evaluate the prospective diagnostic and prognostic values of ESRG in various human cancers.

Materials and methods: The expression of ESRG in various cancers was analyzed using the Gene Expression Profiling Interactive Analysis (GEPIA), Tumor Immune Estimation Resource (TIMER), and University of Alabama at Birmingham Cancer Data Analysis Portal (UALCAN) databases. Moreover, the correlation between the expression of ESRG and clinical pathological parameters was analyzed using UALCAN. The effect of ESRG expression on the survival outcome was evaluated using Kaplan-Meier plotter, UALCAN, GEPIA, and TIMER. The correlation between ESRG expression and immune cell infiltration was studied by TIMER. Additionally, the genetic alterations were investigated cBioportal. Our findings were validated using the GEO2R database.

Results: Our results showed ESRG to be significantly up-regulated in colon adenocarcinoma (COAD) and lung squamous cell carcinoma (LUSC) with p<0.001, in addition to rectum adenocarcinoma (READ), and uterine carcinosarcoma (UCEC) with p<0.01. Regarding pathogenic stages, there was a significant upregulation in stages 2, 3, and 4 compared to normal in COAD and stages 1, 2, and 3 for LUSC patients. The combined prognostic analysis showed that the up-regulated expression of ESRG was associated with better survival outcomes in patients with brain lower-grade glioma (LGG). Our results demonstrate a significant negative correlation between ESRG expression and the abundance of CD8+T cells in COAD, READ, LUSC, and UCEC. Additionally, ESRG was mutated in 0.77 (<1%) of the queried samples, and the most prevalent ESRG mutations are deep deletion mutations, followed by amplification.

Conclusion: Analysis of ESRG across various cancer types elucidated its potential to be used as a diagnostic biomarker in COAD, LUSC, READ, and UCEC and a promising prognostic biomarker in LGG. Our findings provide useful insights for future research.

## Introduction

Embryonic stem cell-related gene (ESRG; also known as HESRG) is a long non-coding RNA (lncRNA) located at chromosome 3p14.3 with a full-length mRNA of 3151 nucleotides consisting of four exons and three introns, found in the nuclei of human embryonic stem cells (hESCs) [1,2]. As an lncRNA, ESRG contains an open reading frame that encodes small peptides, through which it regulates human pluripotent stem cells' (hPSCs) self-renewal ability; as a part of a transcriptional hierarchy in cooperation with many other genes, and is considered to be indispensable for cell survival and self-renewal/pluripotency of hPSCs [3-6].

The level of ESRG expression is not the same in all cells; earlier studies found ESRG to only be exclusively expressed in undifferentiated hESCs, where the expression levels decrease or diminish after differentiation, in addition to being scarcely detected or absent in most adult tissues [1]. Conversely, more recent studies showed that ESRG is expressed in adult tissues like ovary tissues and fibroblasts, however with lower levels than that in pluripotent cells [2,7].

Over the past decades, significant progress has been made in unraveling the molecular mechanisms underlying cancer development and progression. One area of research that has gained attention is the relationship of lncRNA deregulation with cancers and cancer metastasis, this deregulation was found to be related to treatment resistance and poor prognosis in cancers [8,9]. Additionally, previous studies showed that lncRNA expression was proportional to antisense coding gene expression, which is associated with cancers and many other diseases [10]. ESRG gene was proposed to have tumor suppressive effect; relating to its interaction with chromatins, different RNA types, and RNA binding proteins (RBP), which are viewed as critical elements in posttranscriptional gene regulation [11]. Hence, ESRG is considered an interesting gene to study; given that those interactions dictate cell behavior and subsequently the susceptibility to turn cancerogenic, making ESRG to be considered as an oncogenic lncRNA [12-14]. Previous studies have shown that lncRNAs were expressed in various tumor tissues such as breast cancer, thyroid cancer, colorectal cancer, and gastrointestinal cancer, with the potential to be used as prognostic or diagnostic biomarkers [8,13,15,16]. However, the potential of ESRG as a biomarker has been stated by just one study concerning intracranial germinoma and embryonal carcinoma [17]. In this study, we have conducted a comprehensive pan-cancer analysis of ESRG expression including clinicopathological correlation, immune infiltration, and genetic alterations to determine the diagnostic and prognostic value of ESRG using various databases.

## Materials and methods

ESRG expression analysis

The Tumor Immune Estimation Resource (TIMER) 2.0 database (http://timer.cistrome.org/) is an online platform that was used to estimate ESRG differential expression between tumor and normal tissues from The Cancer Genome Atlas (TCGA) database in the “Gene_DE” module [18]. The Gene Expression Profiling Interactive Analysis (GEPIA) (http://gepia.cancer-pku.cn/) database (accessed in 2024) is an online tool used to indicate gene expression from 9736 tumor samples and 8587 normal samples from TCGA and GTEx. ESRG expression was estimated across various cancers using a cutoff of 0.05 for the p-value and 1.5 for the log2FC [19]. Furthermore, the University of Alabama at Birmingham Cancer Data Analysis Portal (UALCAN) database (https://ualcan.path.uab.edu/) is an online resource for analyzing and exploring cancer data from the TCGA database was used to analyze the significance of ESRG differential expression. Moreover, this database was used to investigate the correlation between ESRG expression and clinicopathological parameters analysis including, stage, race, gender, weight, and age [20].

Survival outcome analysis of HESRG across various cancer types

The Kaplan Meier plotter (https://kmplot.com/analysis) is capable of assessing the correlation between the expression of all genes and survival in 35k+ samples from 21 tumor types [21]. The prognostic potential of the ESRG was assessed using this database. Hazard ratios and p-values or log-rank p-values were used for exploring the significance of overall survival (OS), and relapse-free survival (RFS). Moreover, the UALCAN database provides graphs and plots depicting patient survival information for lncRNA-coding genes [20]. Additionally, GEPIA and TIMER databases were used for the same purpose [19].

Immune infiltrates analysis of HESRG across various cancer types

The gene module of TIMER2.0 was used to visualize the correlation of HESRG expression with six immune infiltrates (B cells, CD4+ T cells, CD8+ T cells, neutrophils, macrophages, and dendritic cells) across colon adenocarcinoma (COAD), lung squamous cell carcinoma (LUSC), rectum adenocarcinoma (READ), uterine carcinosarcoma (UCEC), and lower-grade glioma (LGG). Then Cox proportional hazard model of the TIMER database (survival module) was used to explore the association between clinical factors (age and stage) and abundance of six immune infiltrates, and gene expression [18].

Genetic alterations analysis using the cBioPortal platform

The cBio Cancer Genomics Portal (https://www.cbioportal.org/) is an open-access tool for investigating and exhibiting genetic variations using cancer genomic datasets [22]. We applied it to identify the genetic alterations of ESRG, currently offering the data from 10,967 tumor samples over 32 cancer studies, particularly TCGA Pan-Cancer Atlas Studies.

Validation of ESRG expression

We used publicly available datasets from the National Centre for Biotechnology Information (NCBI) (https://www.ncbi.nlm.nih.gov/) to verify our findings. Using the GEO2R program (https://www.ncbi.nlm.nih.gov/geo/geo2r/) [23], an interactive online tool that lets researchers compare at least two groups of samples to find genes that are expressed differently, we carried out differential expression analysis. As a result, we were able to determine the importance of ESRG in COAD, LUSC, READ, UCEC, and LGG. The profile of differentially expressed genes (DEGs) was visualized using volcano plots from the (http://www.bioinformatics.com/.cn) [24] platform, which is an online tool utilized for data visualization and analysis.

## Results

ESRG expression analysis

**Figure 1:**
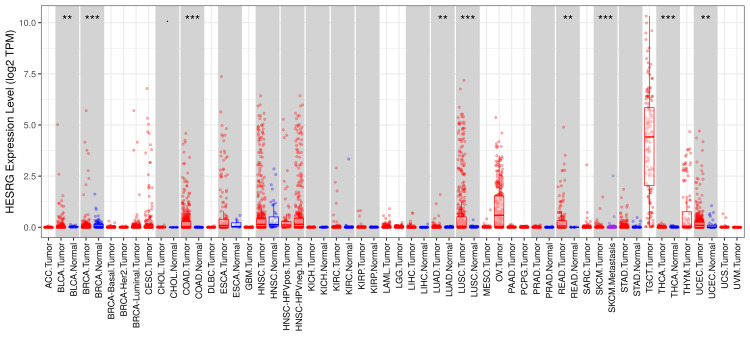
ESRG expression analysis in various tumors using TIMER2.0 database The red columns represent the tumor tissues and the blue ones represent the normal tissues while the stars indicate the differential significance between the tumor and normal samples. *p < 0.05, **p < 0.01 and ***p < 0.001. HESRG: embryonic stem cell-related gene; TIMER: Tumor Immune Estimation Resource; GEPIA: Gene Expression Profiling Interactive Analysis; p: p-value; ACC: adrenocortical carcinoma; BLCA: bladder urothelial carcinoma; BRCA: breast invasive carcinoma; Her2: human epidermal growth factor receptor 2; LumA: luminal A; LumB: luminal B; CESC: cervical squamous cell carcinoma and endocervical adenocarcinoma; CHOL: cholangiocarcinoma; COAD: colon adenocarcinoma; DLBC: lymphoid neoplasm diffuse large B-cell lymphoma; ESCA: esophageal carcinoma; GBM: glioblastoma multiforme; HNSC: head and neck squamous cell carcinoma; HPV+: human papillomavirus positive; HPV-: human papillomavirus negative; KICH: kidney chromophobe; KIRC: kidney renal clear cell carcinoma; KIRP: kidney renal papillary cell carcinoma; LAML: acute myeloid leukemia; LGG: brain lower grade glioma; LIHC: liver hepatocellular carcinoma; LUAD: lung adenocarcinoma; LUSC: lung squamous cell carcinoma; MESO: mesothelioma; OV: ovarian serous cystadenocarcinoma; PAAD: pancreatic adenocarcinoma; PCPG: pheochromocytoma and paraganglioma; PRAD: prostate adenocarcinoma; READ: rectum adenocarcinoma; SARC: sarcoma; SKCM: skin cutaneous melanoma; STAD: stomach adenocarcinoma; TGCT: testicular germ cell tumors; THCA: thyroid carcinoma; THYM: thymoma; UCEC: uterine corpus endometrial carcinoma; UCS: uterine carcinosarcoma; UVM: uveal melanoma

The analysis of the ESRG expression using the TIMER2.0 database (Figure 1) showed a significant upregulation in breast invasive carcinoma (BRCA), COAD, and LUSC with p<0.001, in addition to bladder urothelial carcinoma (BLCA), lung adenocarcinoma (LUAD), READ, and UCEC with p<0.01. Although ESRG expression was significant in glioblastoma multiforme (GBM) and thyroid carcinoma (THCA) with p<0.001, the results do not show whether the gene is upregulated or downregulated in a clear way. Moreover, the expression of ESRG in skin cutaneous melanoma (SKCM) tumor tissues was found to be significant with p<0.001. Due to the absence of some normal tissue samples in the TIMER2.0 database, further analysis of ESRG expression using the GEPIA database was done. The result showed a significant upregulation in testicular germ cell tumors (TGCT) only (see Figure 11 in Appendices).

**Figure 2:**
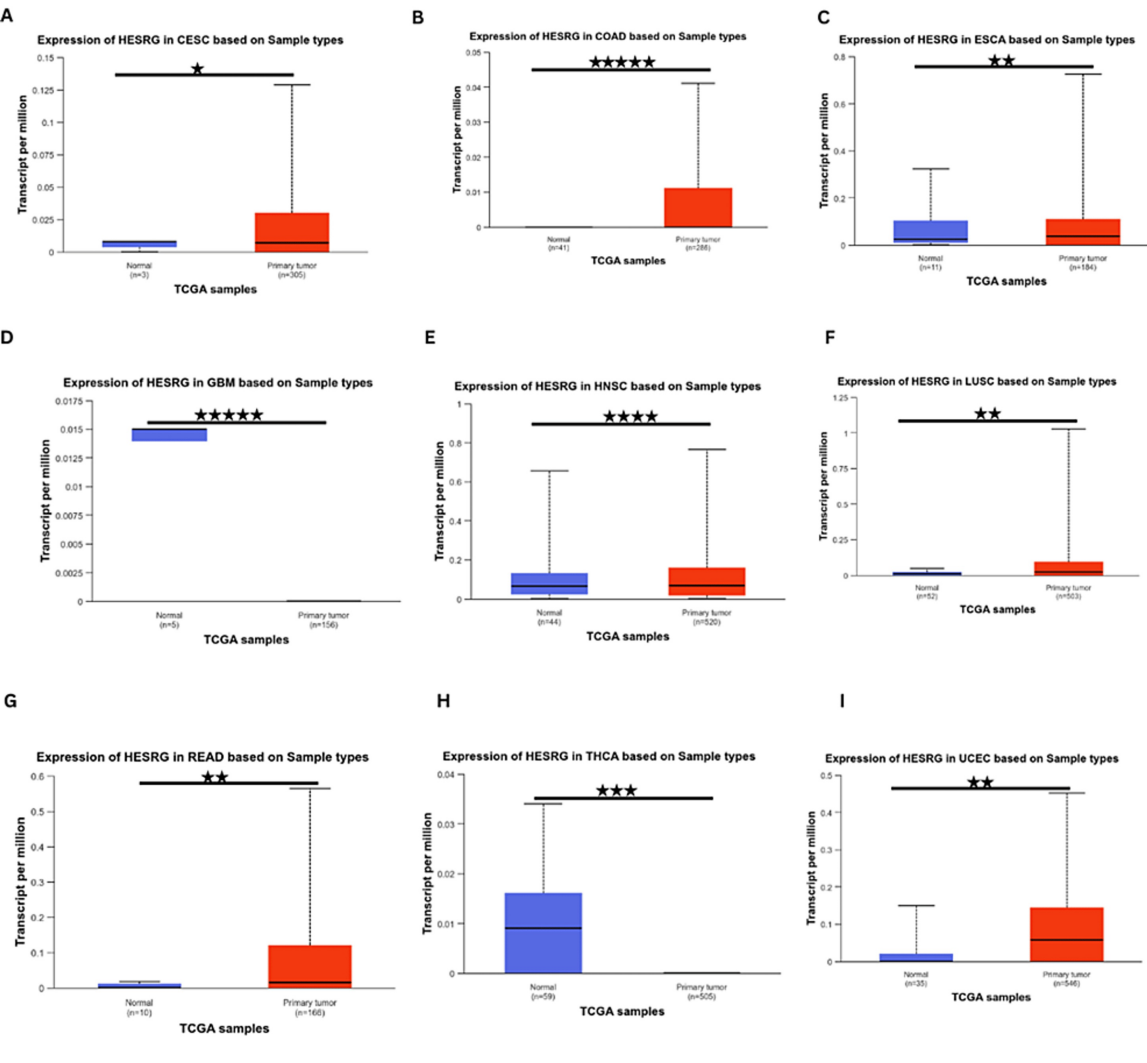
ESRG expression analysis in various tumors using the UALCAN database (A) in CESC, (B) in COAD, (C) in ESCA, (D) in GBM, (E) in HNSC, (F) in LUSC, (G) in READ, (H) in THCA, (I) in UCEC. *p < 0.05, **p < 0.01, ***p < 0.001 and **** p < 0.0001. HESRG: embryonic stem cell-related gene; UALCAN: University of Alabama at Birmingham Cancer Data Analysis Portal; p: p-value; CESC: cervical squamous cell carcinoma and endocervical adenocarcinoma; COAD: colon adenocarcinoma; ESCA: esophageal carcinoma; GBM: glioblastoma multiforme; HNSC: head and neck squamous cell carcinoma; LUSC: lung squamous cell carcinoma; READ: rectum adenocarcinoma; THCA: thyroid carcinoma; UCEC: uterine corpus endometrial carcinoma; n: the number of samples

Further analysis using the UALCAN database showed that ESRG was significantly upregulated in cervical squamous cell carcinoma (CESC), COAD, esophageal carcinoma (ESCA), LUSC, READ, uterine corpus endometrial carcinoma (UCES), head and neck squamous cell carcinoma (HNSC). In contrast, it was significantly downregulated in thyroid carcinoma (THCA) and GBM (Figure 2). Whereas ESRG expression was not significant in adrenocortical carcinoma (ACC), bladder urothelial carcinoma (BLCA), kidney chromophobe (KICH), kidney renal papillary cell carcinoma (KIRP), acute myeloid leukemia (LAML), brain LGG, lung adenocarcinoma (LUAD), liver hepatocellular carcinoma (LIHC), mesothelioma (MESO), sarcoma (SARC), stomach adenocarcinoma (STAD), ovarian serous cystadenocarcinoma (OV), pancreatic adenocarcinoma (PAAD), prostate adenocarcinoma (PRAD), pheochromocytoma and paraganglioma (PCPG), esophageal carcinoma (ESCA), lymphoid neoplasm diffuse large B-cell lymphoma (DLBC), thymoma (THYM), cholangiocarcinoma (CHOL), uterine carcinosarcoma (UCS) and uveal melanoma (UVM) in any of the three databases. By cross-referencing the results from TIMER and UALCAN databases, ESRG was found to be significantly upregulated in COAD, LUSC, READ, and UCEC.

ESRG expression is correlated with clinicopathological parameters

The expression of ESRG was further investigated, by evaluating the association between ESRG expression and clinicopathological parameters including stage, age, race, gender, and weight in COAD, LUSC, READ, and UCEC using UALCAN.

**Figure 3:**
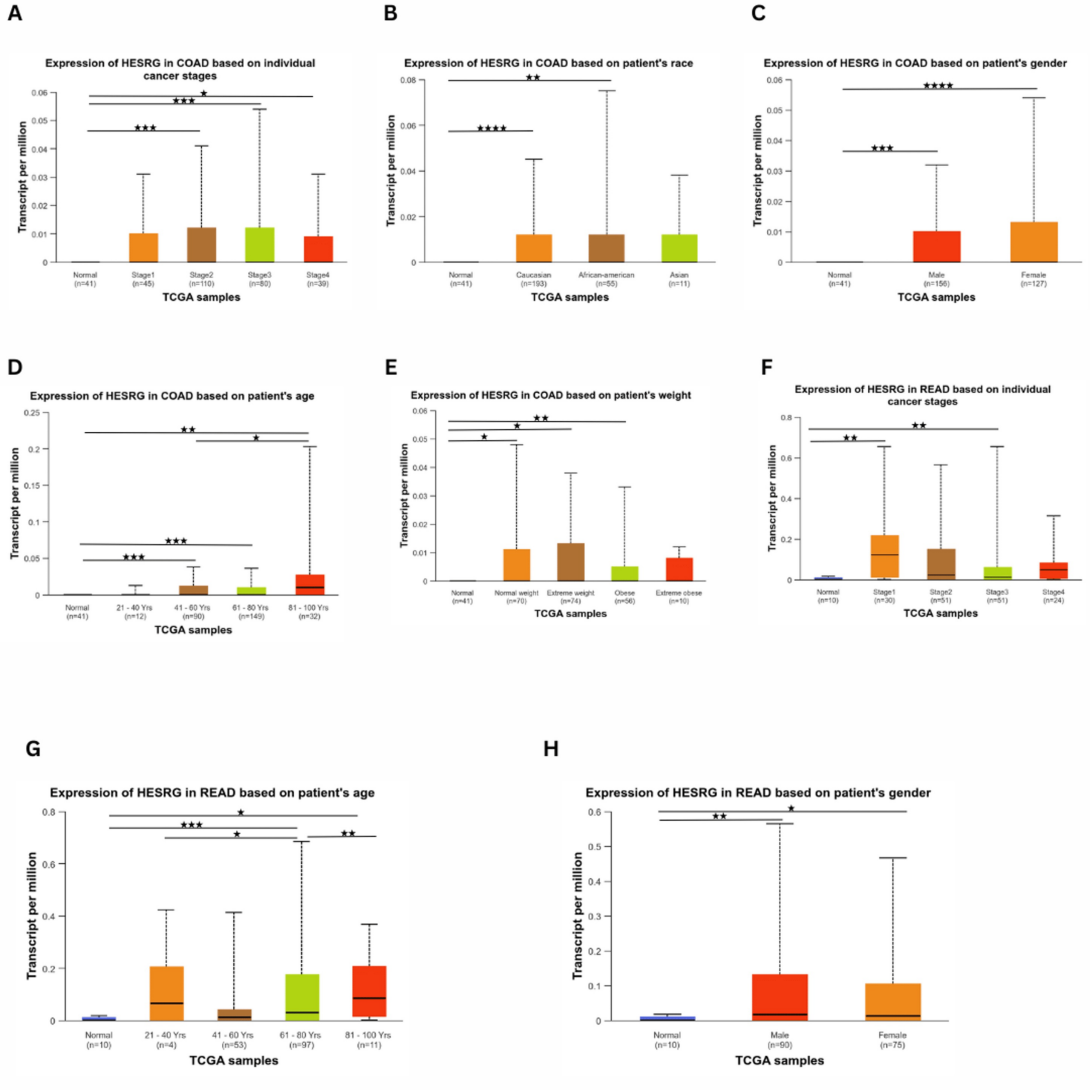
Correlation between HESRG gene expression analysis with clinicopathological features (stage, gender, race, age, and weight) in COAD and READ using UALCAN (A) Expression in COAD based on stage; (B) Expression in COAD based on race; (C) Expression in COAD based on gender; (D) Expression in COAD based on age; (E) Expression in COAD based on weight; (F) Expression in READ based on stage; Expression in READ based on age; (G) Expression in READ based on gender. *p < 0.05, **p < 0.01, ***p < 0.001, ****p < 0.0001 and *****p < 0.00001. HESRG: embryonic stem cell-related gene; UALCAN: University of Alabama at Birmingham Cancer Data Analysis Portal; p: p-value; COAD: Colon adenocarcinoma; READ: Rectum adenocarcinoma; n: number of samples

Regarding expression levels in COAD, the gene expression was significant in stage 2 (p = 2.37e-04), stage 3 (p = 7.44e-04), and stage 4 (p = 1.188e-02) compared to normal (Figure 3A). As for race, the gene expression was higher than normal in Caucasian (p = 2.24e-05) and African American (p = 2.58e-03) patients, while being insignificant in Asians (Figure 3B). There was a significant expression difference between normal and tumorous tissues in males (p = 4.80e-04) and females (p = 5.64e-05) (Figure 3C). The expression of ESRG was upregulated at age 41-60 (p = 9.19e-04), age 61-80 (p = 8.597e-04) and age 81-100 (p = 6.56e-03). In addition, there was a significant difference in expression between age 41-60 and age 81-100 (p = 4.39e-02), with no significant difference between normal and younger patients (Figure 3D). Based on weight, the expression of ESRG was significant in patients with normal weight (p = 1.60e-02), extreme weight (p = 9.58e-03), and obesity (p = 8.70e-03) (Figure 3E).

For READ, ESRG was upregulated in stage 1 (p = 3.20e-03) and stage 3 (p = 6.36e-03). In contrast, there was no significant difference in stage 2 compared to normal (Figure 3F). Considering age (Figure 3G), ESRG was upregulated in patients 61-80 years (p = 3.80e-04) and 81-100 years (p = 1.02). Also, there was a difference between patients with 21-40 years and 61-80 years (p = 2.29e-02), as well as patients having 61-80 years and 81-100 (p = 5.82e-03). However, there was no significance of patient weight and ESRG expression level compared to normal. Additionally, ESRG expression was higher in males (p = 3.38e-03) and females (p = 2.95e-02) (Figure 3H). However, ESRG expression was not significantly different based on race (Caucasian, African, and Asian).

**Figure 4:**
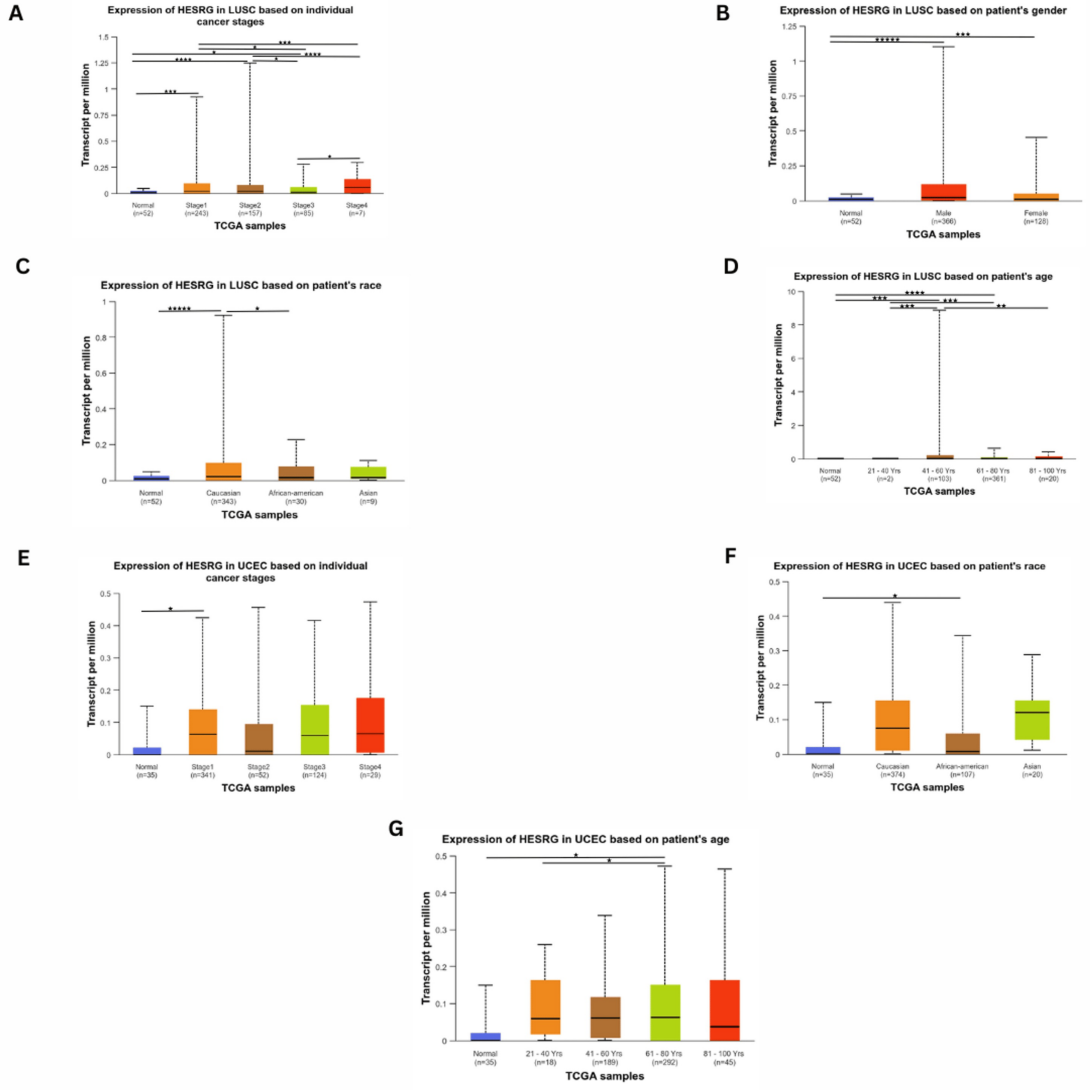
Correlation between HESRG gene expression analysis with clinicopathological features (stage, age, gender, race, and weight) in LUSC and UCEC using UALCAN. (A) Expression in LUSC based on stage; (B) Expression in LUSC based on gender; (C) Expression in LUSC based on race; (D) Expression in LUSC based on age; (E) Expression in UCEC based on stage; (F) Expression in UCEC based on gender; (J) Expression in UCEC based on age. *p < 0.05, **p < 0.01, ***p < 0.001, ****p < 0.0001 and *****p < 0.00001. HESRG: embryonic stem cell-related gene; UALCAN: University of Alabama at Birmingham Cancer Data Analysis Portal; p: p-value; LUSC: Lung squamous cell carcinoma; UCEC: Uterine Carcinosarcoma; n: number of samples

In LUSC patients, the gene expression was significant in stage 1 (p = 1.22e-04), stage 2 (p = 1.54e-05), and stage 3 (p = 1.59e-02). Further, the was a significant difference between stage 1 and stage 3 (p = 2.17e-02), stage 1 and stage 4 (p = 1.75e-04), stage 2 and stage 3 (p = 1.37e-02), stage 2 and stage 4 (p = 1.84e-05) and stage 3 and stage 4 (2.39e-04). The expression is the highest in stage 2 (Figure 4A). There was a significant difference between normal and tumorous tissues in males (p = 9.26e-07) and females (p = 3.04e-04) (Figure 4B). Moreover, the gene expression was higher than normal in Caucasians (p = 1.02e-06), and not significant in Africans and Asians. However, there was a significant difference between Caucasian and African (p = 2.14e-02) patients (Figure 4C). As for weight, there was no significant difference in ESRG expression. Regarding age (Figure 4D), the expression of ESRG was higher in age 41-60 (p = 3.15e-04) and age 61-80 (p = 2.26e-04) than normal. Furthermore, there was a significant difference in expression between age 21-40 and age 41-60 (p = 3.16e0-4), age 21-40 and age 61-80 (p = 2.28e-05) and also between age 41-60 and 61-80 (p = 9.68e-03).

As for UCEC patients, ESRG expression was only significant in stage 1 (p = 3.31e-02), with no significant difference in other stages (Figure 4E). It was also found to be upregulated in African Americans (p = 2.91e-02), and not significant in Caucasians and Asians (Figure 4F). ESRG expression was significant in patients with 61-80 years (p = 1.14e-02) compared to patients who are younger and older than this age range, where the expression difference was not significant compared to normal. However, there was a difference in expression between patients aged 21-40 and 61-80 years (p = 1.05e-02) (Figure 4G). Meanwhile, there was no significant difference in various patients' weight range and ESRG expression levels compared to normal.

The correlation between ESRG expression and survival outcome in various cancers

**Figure 5:**
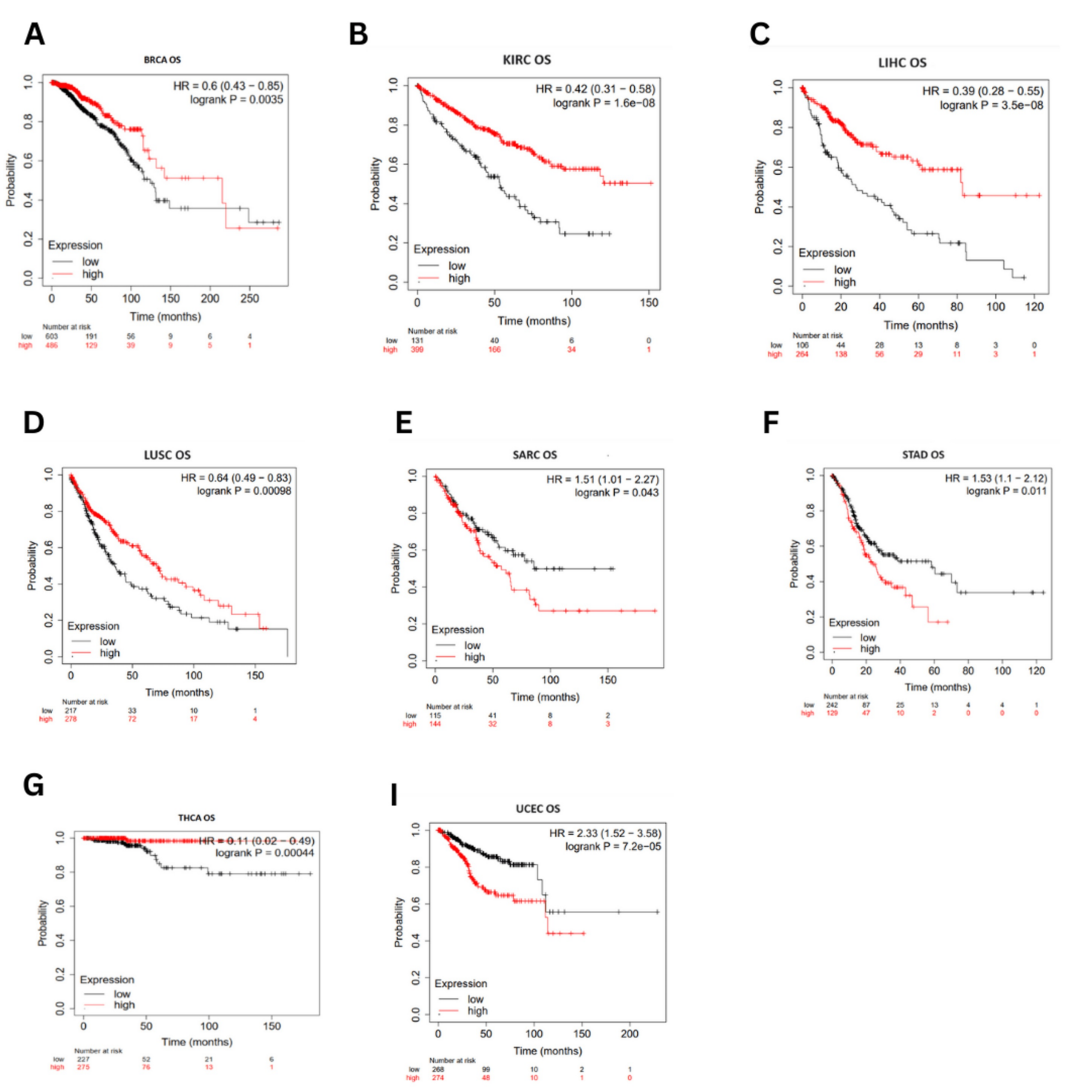
The correlation between ESRG expression and OS in different cancers using Kaplan-Meier plotter The red line represents high gene expression and the black line represents low gene expression. p < 0.05. ESRG: embryonic stem cell-related gene; HR: hazard ratio, logrank P: p-value resulting from logrank test; OS: overall survival; SARC: sarcoma; LUSC: lung squamous cell carcinoma; LIHC: liver hepatocellular carcinoma; BRCA: breast invasive carcinoma; UCEC: uterine corpus endometrial carcinoma; THCA: thyroid carcinoma; KIRC: kidney chromophobe; STAD: stomach adenocarcinoma

Survival correlation to ESRG expression was analyzed using the Kaplan-Meier plotter database (accessed in March 2024). Higher expression of ESRG was associated with better OS in BRCA (OS: HR = 0.6, p = 0.0035) LIHC (OS: HR = 0.39, p = 3.5E-08), LUSC (HR = 0.64, p = 0.00098), KIRC (HR = 0.42, p = 1.6E-08) and THCA (HR = 0.11, p = 0.00044). On the other hand, OS was poorer in STAD (OS: HR = 1.53, p = 0.011), SARC (OS: HR = 1.51, p = 0.043), and UCEC (OS: HR = 2.3, p = 7.2E-05) (Figure 5).

**Figure 6:**
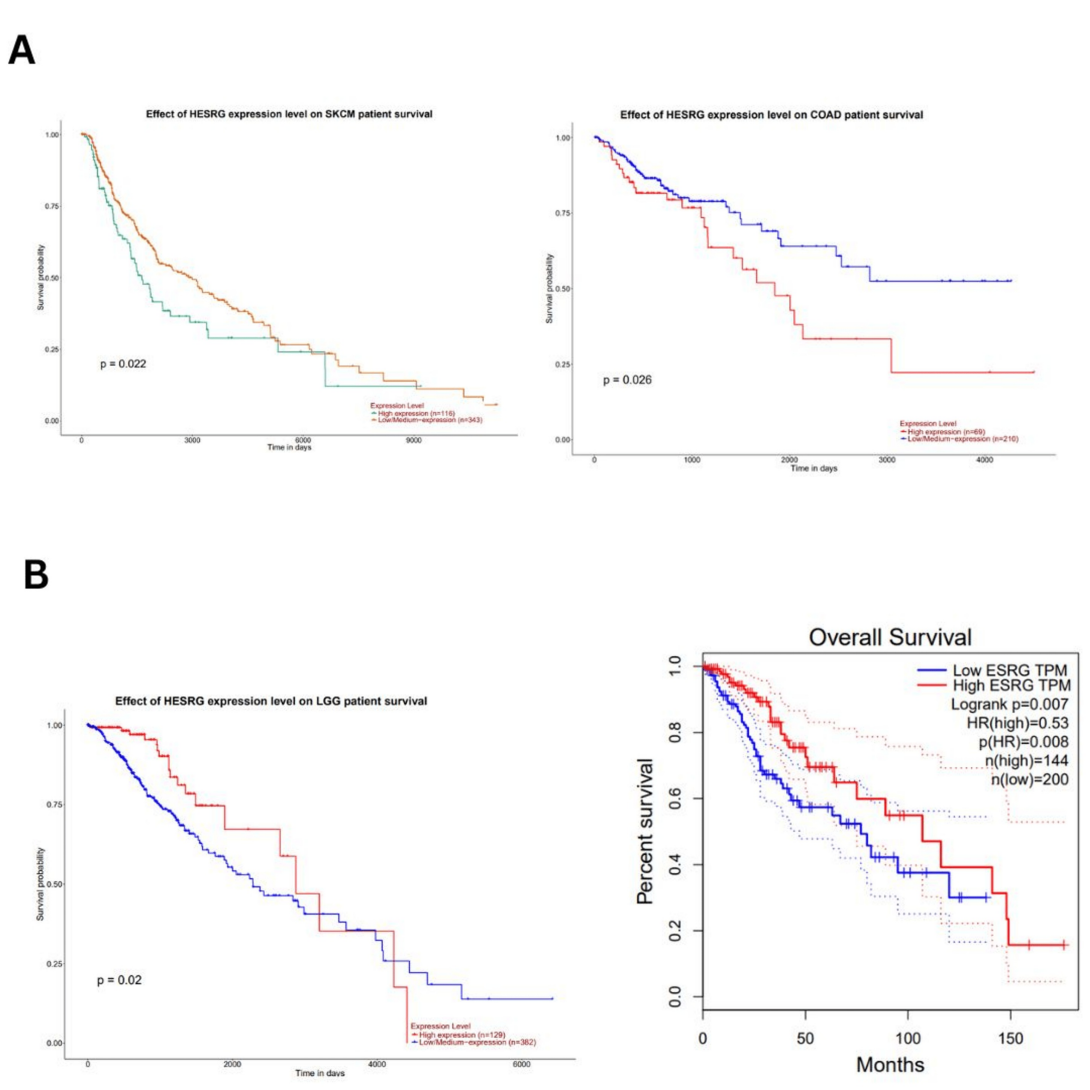
The correlation between ESRG expression and survival outcome (A) The correlation between ESRG expression and survival outcome in SKCM and COAD using UALCAN. (B) The correlation between ESRG expression and survival outcome in LGG using GEPIA and UALCAN. HR: hazard ratio; logrank P: p-value resulting from logrank test; p: p-value, HESRG/ESRG: embryonic stem cell-related gene; COAD: colon adenocarcinoma; SKCM: skin cutaneous melanoma gene; LGG: brain lower grade glioma; TPM: transcripts per million

UALCAN database (accessed in March 2024) was also used to analyze the survival outcome of ESRG, we found that high ESRG expression was associated with poor survival in COAD (p = 0.026), and SKCM (p = 0.022) as illustrated in (Figure 6A), while it's associated with better survival in LGG patients (p = 0.02) (Figure 6B). Further analysis by the GEPIA database (accessed on March 23, 2024) showed that high ESRG expression was associated with longer overall survival in LGG patients (OS: HR = 0.53, p = 0.008; RFS: HR = 0.62, p = 0.018) (Figure 6B). By cross-referencing the results confirmed between two databases UALCAN and GEPIA databases, ESRG was found to be significant in LGG with better survival (Figure 6B).

The correlation between ESRG expression and immune infiltration

Using the TIMER2.0 database, we analyze tumor-infiltrating immune cells over 10,000 RNA seq. samples across 23 cancer types from TCGA. The correlation between ESRG expression and the abundance of immune cells was investigated in COAD, LUSC, READ, UCEC, and LGG.

**Figure 7:**
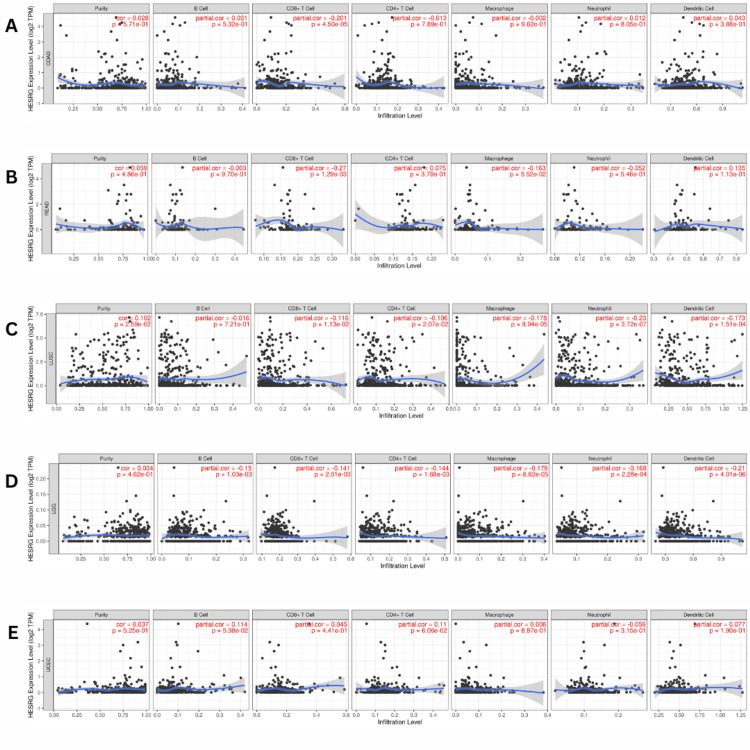
The correlation between HESRG expression and the abundance of immune cells (B cells, CD8+ T cells, CD4+ T cells, macrophages, neutrophils, and dendritic cells, in addition to tumor purity) using the TIMER database. (A) COAD, (B) READ, (C) LUSC, (D) LGG, (E) UCEC. TIMER: Tumor Immune Estimation Resource; HESRG: embryonic stem cell-related gene; COAD: colon adenocarcinoma; LUSC: lung squamous cell carcinoma; READ: rectum adenocarcinoma; UCEC: uterine corpus endometrial carcinoma; LGG: brain lower grade glioma; B cells: B lymphocytes CD8+ T cells: cytotoxic T lymphocytes; CD4+ T cells: T helper cells; Cor: correlation; p: p-value; log2 TPM: logarithm of 2 transcripts per million

In patients with COAD and READ, we found a significant negative correlation between the expression of ESRG and the abundance of CD8+ T cells in COAD (Cor = -0.201, p = 4.50E-05) (Figure 7A) and READ (Cor = -0.027, p = 0.001) (Figure 7B). Additionally, ESRG expression levels were negatively correlated with the infiltration of CD8+ T cells (Cor = -0.116, p =0.01), CD4+ T cells (Cor = -0.106, p = 0.02), macrophages (Cor = -0.178, p =8.94E-05), neutrophils (Cor = -0.230, p =3.72E-07), and dendritic cells (Cor = -0.173, p =0.0001) in LUSC (Figure 7C). While in LGG, there was a negative correlation between the expression of ESRG and the abundance of B cells (Cor = -0.15, p =0.001), CD8+ T cells (Cor = -0.141, p = 0.002), CD4+ T cells (Cor = -0.144, p =0.001), macrophages (Cor = -0.179, p = 8.82E-05), neutrophils (Cor = -0.168, p = 0.0002), and dendritic cells (Cor = -0.209, p = 4.01E-06) (Figure 7D). In contrast, an insignificant correlation between ESRG expression and immune infiltration was seen in patients with UCEC (Figure 7E).

Correlation of ESRG expression with the survival rate of patients with COAD, READ, LUSC, UCEC, and LGG

**Figure 8:**
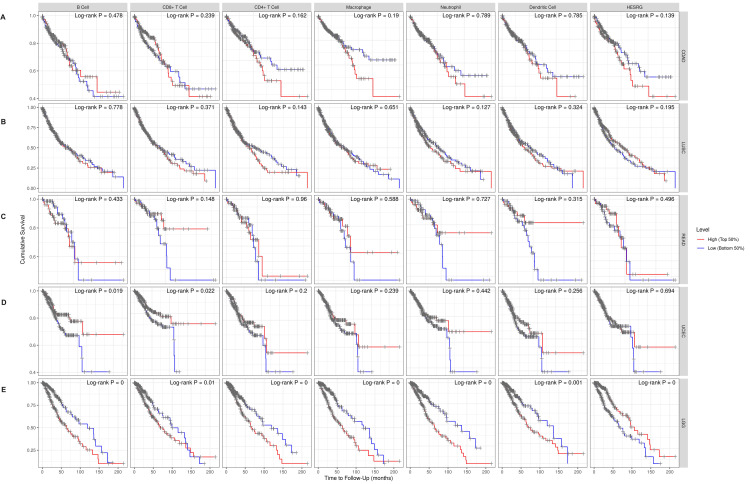
Kaplan-Meier plots exhibited the correlation between ESRG expression, immune cell infiltrates (B cells, CD4+ T Cells, CD8+ T cells, macrophages, neutrophils, and dendritic cells), and the survival outcome of patients with cancer using the TIMER database. (A) COAD, (B) READ, (C) LUSC, (D) UCEC, (E) LGG. TIMER: Tumor Immune Estimation Resource; ESRG: embryonic stem cell-related gene; COAD: colon adenocarcinoma; LUSC: lung squamous cell carcinoma; READ: rectum adenocarcinoma; UCEC: uterine corpus endometrial carcinoma; LGG: brain lower grade glioma; B cells: B lymphocytes; CD8+ T cells: cytotoxic T lymphocytes; CD4+ T cells: T helper cells; logrank P: p-value resulting from logrank test

The survival outcome considering the correlation of ESRG expression with immune cells was investigated using the Cox proportional hazard model of the TIMER database adding the clinical parameters of age and stage (Figure 8). For COAD, LUSC, READ, and UCEC patients, ESRG expression was not significantly correlated with survival (p = 0.139, 0.195, 0.496, and 0.695, respectively). However, high ESRG expression was associated with better survival in patients with LGG (p = 0.00). The Cox proportional hazard model showed that age, stage 3, and stage 4, were associated with poor prognosis in COAD (p-values: age = 0.00, stage 3 = 0.008, stage 4 = 0.00) (Table 1) and LUSC patients (p-values: age = 0.039, stage 3 = 0.012, stage 4 = 0.010) (Table 2). Moreover, the poor prognosis was associated with the age factor (p = 0.006) in READ (Table 3). Age was found to be associated with poor prognosis (p = 0.001) in UCEC, but CD8- T cell (p = 0.003), and CD4- T cell (p = 0.011) were associated with better prognosis (Table 4). While in LGG, age (p = 0.000) and macrophage (p = 0.009) were associated with poor prognosis (Table 5).

**Table 1:** Cox proportional hazard model of HESRG, immune cells, and clinical parameters (age and stages) in COAD patients (433 patients with 94 dying). HESRG: embryonic stem cell-related gene; COAD: colon adenocarcinoma; Coef: coefficient; HR: hazard ratio; 95%CI_l: lower 95% confidence interval; 95%CI_u: upper 95% confidence interval; p: p-value; sig: significance. **p < 0.01, ***p < 0.001.

Factors	Coef	HR	95%CI_l	95%CI_u	p-value	sig
Age	0.039	1.039	1.021	1.059	0.000	***
Stage 2	0.581	1.787	0.677	4.719	0.241	-
Stage 3	1.320	3.745	1.411	9.936	0.008	**
stage4	2.411	11.144	4.139	30.005	0.000	***
B_cell	2.774	16.019	0.136	1893.292	0.255	-
CD8_Tcell	-3.464	0.031	0.001	1.720	0.090	-
CD4_Tcell	-0.569	0.566	0.005	69.425	0.817	-
Macrophage	2.533	12.594	0.090	1754.574	0.315	-
Neutrophil	-2.719	0.066	0.000	100.296	0.467	-
Dendritic	1.215	3.372	0.165	68.767	0.429	-
HESRG	0.381	1.464	1.141	1.877	0.003	**

**Table 2:** Cox proportional hazard model of ESRG, immune cells, and clinical parameters (age and stages) in LUSC patients (472 patients with 203 dying). HESRG: embryonic stem cell-related gene; LUSC: lung squamous cell carcinoma; Coef: coefficient; HR: hazard ratio; 95%CI_l: lower 95% confidence interval; 95%CI_u: upper 95% confidence interval; p: p-value; sig: significance. *p < 0.05

Factors	Coef	HR	95%CI_l	95%CI_u	p.value	sig
Age	0.018	1.018	1.001	1.036	0.039	*
Stage 2	0.132	1.141	0.820	1.588	0.435	-
Stage 3	0.477	1.611	1.109	2.338	0.012	*
Stage 4	1.204	3.332	1.328	8.363	0.010	*
B_cell	1.085	2.959	0.261	33.535	0.381	-
CD8_Tcell	-1.387	0.250	0.043	1.446	0.122	-
CD4_Tcell	0.708	2.029	0.172	23.880	0.574	-
Macrophage	-0.259	0.772	0.071	8.409	0.832	-
Neutrophil	0.811	2.250	0.095	53.055	0.615	-
Dendritic	0.601	1.823	0.451	7.375	0.400	-
HESRG	-0.058	0.944	0.840	1.061	0.333	-

**Table 3:** Cox proportional hazard model of ESRG, immune cells, and clinical parameters (age and stages) in READ patients (159 patients with 23 dying). HESRG: embryonic stem cell-related gene; READ: rectum adenocarcinoma; Coef: coefficient; HR: hazard ratio; 95%CI_l: lower 95% confidence interval; 95%CI_u: upper 95% confidence interval; p: p-value; sig: significance. **p < 0.01

Factors	Coef	HR	95%CI_l	95%CI_u	p.value	sig
Age	0.076	1.079	1.022	1.140000e+00	0.006	**
Stage 2	-0.295	0.745	0.136	4.085000e+00	0.734	-
Stage 3	0.416	1.516	0.298	7.714000e+00	0.616	-
Stage 4	1.420	4.138	0.839	2.041600e+01	0.081	-
B_cell	-2.043	0.130	0.000	8.374687e+03	0.718	-
CD8_Tcell	-14.057	0.000	0.000	1.262530e+02	0.145	-
CD4_Tcell	-9.945	0.000	0.000	1.894461e+05	0.378	-
Macrophage	1.355	3.878	0.000	2.821583e+06	0.844	-
Neutrophil	1.620	5.055	0.000	3.540224e+012	0.907	-
Dendritic	7.906	2714.120	0.087	8.487545e+07	0.134	-
HESRG	-0.201	0.818	0.358	1.868000e+00	0.634	-

**Table 4:** Cox proportional hazard model of ESRG, immune cells, and age in UCEC patients (529 patients with 87 dying). HESRG: embryonic stem cell-related gene; UCEC: uterine corpus endometrial carcinoma; Coef: coefficient; HR: hazard ratio; 95%CI_l: lower 95% confidence interval; 95%CI_u: upper 95% confidence interval; p: p-value; sig: significance. *p < 0.05, **p < 0.01

Factors	Coef	HR	95%CI_l	95%CI_u	p.value	sig
Age	0.035	1.036	1.015	1.057	0.001	**
B_cell	-3.807	0.022	0.000	11.968	0.235	-
CD8_Tcell	-6.610	0.001	0.000	0.111	0.003	**
CD4_Tcell	-8.120	0.000	0.000	0.151	0.011	*
Macrophage	3.768	43.276	0.285	6579.962	0.142	-
Neutrophil	5.482	240.425	0.138	419984.289	0.150	-
Dendritic	2.532	12.580	0.411	384.661	0.147	-
HESRG	0.113	1.119	0.822	1.523	0.474	-

**Table 5:** Cox proportional hazard model of ESRG, immune cells, and age in LGG patients (504 patients with 122 dying). HESRG: embryonic stem cell-related gene; LGG: brain lower grade glioma; Coef: coefficient; HR: hazard ratio; 95%CI_l: lower 95% confidence interval; 95%CI_u: upper 95% confidence interval; p: p-value; sig: significance. **p < 0.01, ***p < 0.001.

Factors	Coef	HR	95%CI_l	95%CI_u	p.value	sig
Age	0.054	1.056	1.039	1.072	0.000	***
B_cell	3.377	29.271	0.127	6750.894	0.224	-
CD8_Tcell	4.826	124.692	0.121	128167.731	0.173	-
CD4_Tcell	-0.192	0.826	0.000	1700.913	0.961	-
Macrophage	5.180	177.594	3.662	8613.772	0.009	**
Neutrophil	-3.917	0.020	0.000	50.411	0.327	-
Dendritic	0.681	1.976	0.050	78.692	0.717	-
HESRG	-8.066	0.000	0.000	8.443	0.121	-

Genetic alterations analysis using cBioPortal platform

**Figure 9:**
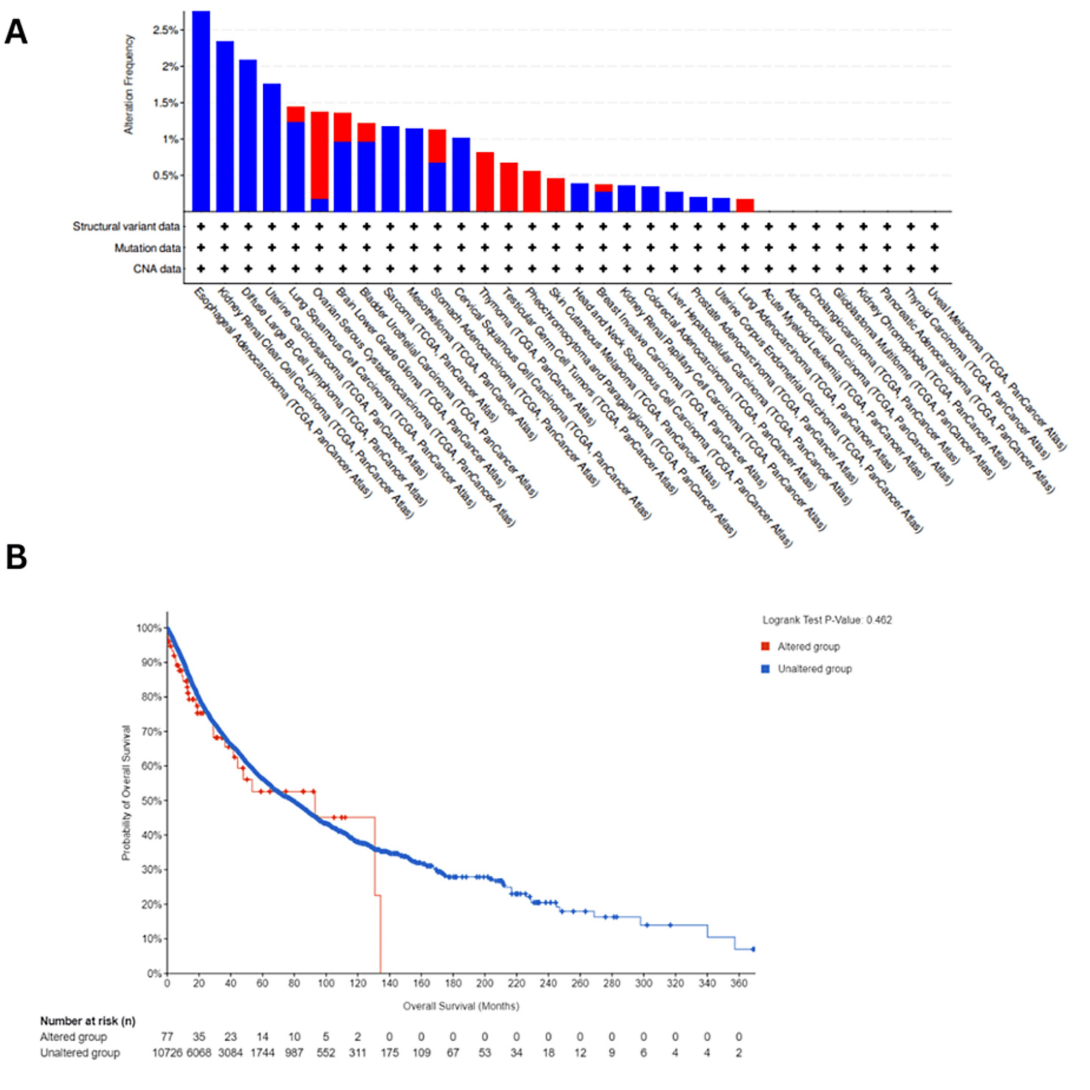
Genetic alterations analysis of ESRG using cBioPortal platform (A) Genetic alterations frequency of ESRG in various cancers. (B) The correlation between the survival outcome of patients with different cancer types and ESRG genetic alterations. ESRG: embryonic stem cell-related gene; TCGA: The Cancer Genome Atlas; CNA: Copy number alterations; logrank P: p-value resulting from log rank test

The genetic alterations in ESRG were analyzed using the cBioPortal platform for cancer genomics using TCGA datasets, we found that ESRG was mutated in 0.77 (<1%) of the queried samples (10,967 samples from 32 studies). The most prevalent ESRG mutations are deep deletion mutations, followed by amplification. We noticed that the majority of ESRG mutations occurred in esophageal adenocarcinoma with deep deletion mutation frequency = 3.3% (six cases) (Figure 9A). Furthermore, we observed that ESRG expression was not mutated in the vast majority of samples, also there was no variation in survival between patients with mutated ESRG and patients with non-mutated ESRG (p= 0.46) as manifested in (Figure 9B).

Validation of ESRG expression

To validate our results of ESRG expression in COAD, LUSC, READ, UCEC, and LGG, four expression profile microarray datasets (GSE87211 for COAD and READ, GSE149507 for LUSC, GSE63514 for UCEC and GSE35493 for LGG) were retrieved from the Gene Expression Omnibus (GEO) database for analysis. Then we applied the GEO2R tool to detect the differential expression of ESRG using (|log2FC| >= 0.2 for COAD and READ, |log2FC| >= 0.1 for LUSC and LGG, |log2FC| >= 3.0 for UCEC) and (p-value < 0.05).

For the GSE87211 dataset, the normal group encompassed 160 control mucosa and 203 rectal tumor samples, whereas GSE149507 contained 18 normal lung and 18 small cell lung cancer samples, while nine normal brain and 33 brain tumor samples were included in GSE35493. For GSE63514, 24 normal cervical epithelium and 28 cervical squamous epithelial cancer samples were included.

**Figure 10:**
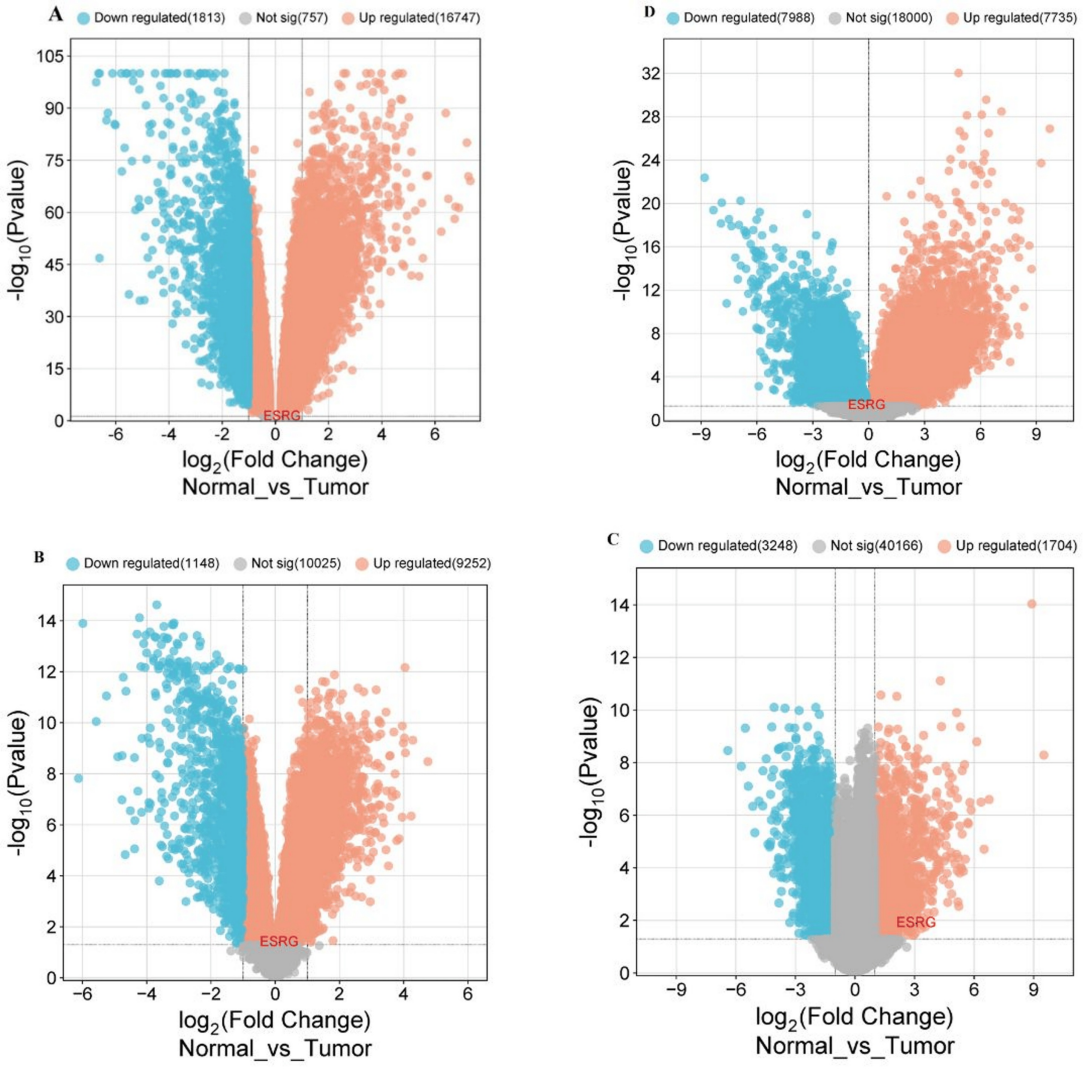
Volcano plots demonstrate differential gene expression, including ESRG, in (A) COAD and READ, (B) LUSC, (C) UCEC, (D) LGG using the http://www.bioinformatics.com/ platform. ESRG: embryonic stem cell-related gene; COAD: colon adenocarcinoma; LUSC: lung squamous cell carcinoma; READ: rectum adenocarcinoma; UCEC: uterine corpus endometrial carcinoma; LGG: brain lower grade glioma; Log2FC: log2FoldChange; GSE87211: Gene Expression Omnibus Series with a unique identifier for colorectal cancer datasets; GSE149507: Gene Expression Omnibus Series with a unique identifier for lung cancer datasets, GSE63514:Gene Expression Omnibus Series with a unique identifier for cervical cancer datasets and GSE35493: Gene Expression Omnibus Series with a unique identifier for brain tumor

**Figure 11:**
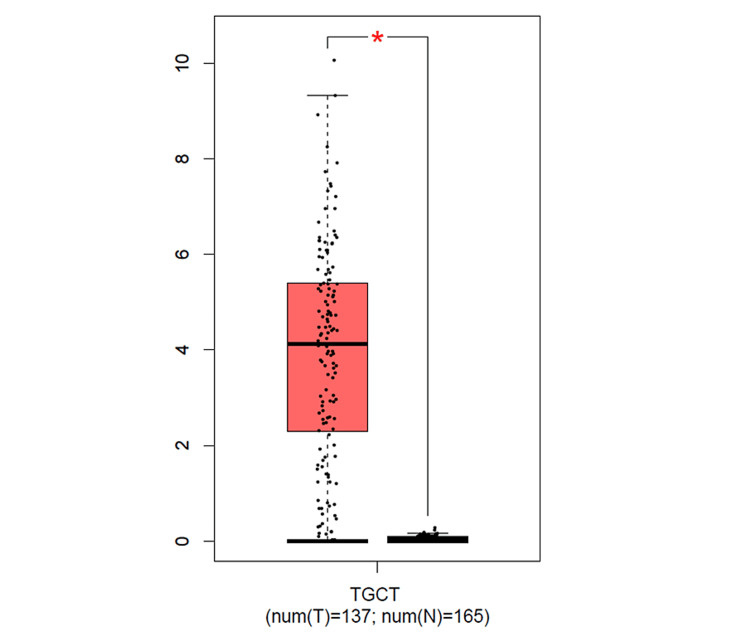
GEPIA expression analysis of HESRG between TCGA tumor tissues and GTEx database normal tissue *p < 0.05 HESRG: embryonic stem cell-related gene; GEPIA: Gene Expression Profiling Interactive Analysis; TCGA: The Cancer Genome Atlas; p: p-value; TGCT: testicular germ cell tumors; num(T): the number of tumor samples; num(N): the number of normal samples

Our results demonstrate that ESRG was upregulated in COAD and READ (|log2FC| = 0.249, p-value = 0.0699), LUSC (|log2FC| = 0.123, p-value = 0.0411), UCEC (|log2FC| = 3.078, p-value = 0.013), while being down-regulated in LGG (|log2FC| = -0.107, p-value = 0.0462). Additionally, our findings demonstrate variable numbers of DEGs including downregulated and upregulated genes for cancers under study as illustrated in Table 6. After that, the volcano plot was produced using the https://www.bioinformatics.com.cn/ online platform to visualize the DEGs using fold change threshold 0.5 for COAD, READ, and LUSC, 2.0 for UCEC and 1 for LGG (Figure 10).

**Table 6:** Number of differentially expressed genes (upregulated and downregulated genes) in four GEO datasets GEO: Gene Expression Omnibus; DEGs: differentially expressed genes

GEO accession	GSE87211	GSE87211	GSE63514	GSE35493
DEGs	18560	10400	4952	15723
Upregulated	16747	9252	1704	7735
Downregulated	1813	1148	3248	7988

## Discussion

The paper investigates the role of ESRG, a lncRNA in various cancers. We employed multiple bioinformatics tools and databases to analyze ESRG expression levels, their correlation with clinicopathological parameters and immune cell infiltrations, prognostic value, and genetic alterations across different cancer types.

We employed GEPIA, TIMER, and UALCAN databases to identify any notable variations of ESRG expression across a range of malignancies considering the findings that were consistent between TIMER and UALCAN databases. Our findings revealed significant ESRG upregulation in COAD, LUSC, READ, and UCEC; hence, it can be used as a potential diagnostic biomarker to distinguish normal from tumor samples. The significant upregulation in COAD and READ aligns with the findings of another study which revealed that ESRG showed an aberrant upregulation in colorectal cancer using quantitative polymerase chain reaction (qPCR) and explored a negative correlation with overall survival [11]. Regarding LUSC, there is a study establishing the association between ESRG overexpression and resistance to chemotherapy implying the gene regulatory networks (GRNs) [25]; this is due to the fact that ESRG as lncRNAs have been proven to contribute to anticancer therapy resistance [26], and the existence of the ESRG in the GRN emphasizes the presence of cancer stem cells in the cancer population, which are known to induce resistance to chemotherapy [27]. To our knowledge, there was no published data to demonstrate the relationship between ESRG expression and UCEC.

Based on our findings, the significant upregulation of ESRG in these cancers COAD, LUSC, READ, and UCEC implies its role in carcinogenesis. However, the association between ESRG expression and cancer is still being studied. The expression of ESRG in the cancer cell population might indicate the presence of cancer stem cell potentials [27]; this has been further justified by its critical role in sustaining pluripotency and self-renewal capacity in hPSCs through numerous mechanisms [1]. One study stated that ESRG acts as a novel octamer-binding factor transcription factor 4 OCT4 target that works with minichromosome maintenance protein 2 (MCM2) to decrease tumor protein p53 signaling [6]. Another study reported that ESRG is bound to and stabilizes heterogeneous nuclear ribonucleoprotein A1 (HNRNPA1) using the ubiquitin-proteasome system [28]. Furthermore, findings suggested that, by its interaction with cytochrome c oxidase subunits II COXII, ESRG may be crucial in controlling the apoptosis of hESC and thus significantly contributing to the preservation of hESC properties [29]. However, a previous study found that cells retained their regeneration and self-renewal abilities despite the knock-out of the ESRG gene, and they suggest that the role of ESRG can be restrained to being a biomarker for pluripotency [30].

Furthermore, the UALCAN database was used to examine the correlation between ESRG expression and clinicopathological parameters. Our results highlight significant variations in the terms of age for UCEC and for both age and gender for COAD, LUSC, and READ. Regarding race, ESRG was significantly upregulated in Caucasians in both COAD and LUSC, in African Americans in COAD and UCEC, and also in Africans in LUSC. Considering weight ESRG is being upregulated significantly just in COAD. Moreover, our findings figured out relatively significant variations in ESRG expression between normal and different cancer stages in COAD, LUSC, and READ. However, in UCEC, ESRG was significantly expressed only in stage 1. As far as we know this study is the first to demonstrate the significant association between ESRG expression and clinicopathological parameters. However, these significant variations within cancer stages may explore the potential role of ESRG in tumor progression which needs to be further studied.

The prognostic analysis was done using three databases: TIMER, UALCAN, and GEPIA to investigate the correlation between ESRG expression and survival, considering the results that are in line with the three databases: TIMER, UALCAN, and GEPIA. It was discovered that the higher expression level of the ESRG gene was associated with a good prognosis in LGG. So these findings indicate a significant interaction between ESRG expression and patient survival, and its potential to be used as a prognostic biomarker in LGG. Comparing our study findings to existing literature there is one study that examined the expression of ESRG in various intracranial malignancies and stated that ESRG was only expressed in embryonal carcinoma and germinoma but barely in the other forms of brain tumors and concluded that the ESRG gene as a sensitive biomarker for these tumors [17].

On top of that, the study demonstrates the association between ESRG expression and immune cell infiltrations across various cancers including COAD, LUSC, READ, UCEC, and LGG. Our findings indicate weak negative correlations between ESRG expression and the abundance of CD8+ T cells in COAD, LUSC, READ, and LGG, also with CD4+ T cells, macrophages, neutrophils, and dendritic cells in LUSC and LGG. Additionally, with B cells in LGG, this significant correlation implies the potential immunosuppressive effects of ESRG, contributing to immune evasion and tumor progression. However, the precise mechanisms underlying this association require further investigation.

Additionally, genetic alterations analysis using the cBioPortal platform revealed that ESRG mutations are rare across different cancer types, with deep deletion mutations being the most prevalent. However, the impact of ESRG mutations on patient survival appears to be minimal, suggesting that other factors may predominantly influence ESRG-mediated carcinogenesis.

Our research is the first pan-cancer analysis of ESRG. It provides a comprehensive analysis to elucidate the correlation between ESRG expression and its role in cancer development and progression across various types of cancers. Our results provide a foundation for exploring the association between ESRG expression and the abundance of immune cells considering their complex interaction with patients' survival in different cancer types to be further investigated. These results can be the base of using ESRG expression as a biomarker that can be used in diagnosis and monitoring of the cancers discussed in the study.

The study has several limitations; firstly, the analysis depends on bioinformatics tools and publicly accessible datasets, so experimental wet lab analysis is required to verify our findings. Additionally, the functional roles of ESRG in immune regulation and cancer progression remain incompletely understood and need further investigation. Moreover, the study primarily focuses on ESRG expression levels and their association with clinical outcomes in different cancers, ignoring the potential regulatory mechanisms and interactions with other molecular pathways. Finally, there are a limited number of datasets for uterine carcinoma and our gene was not present in differentially expressed genes obtained from GEO2R analysis, so we used cervical carcinoma datasets to validate our findings of uterine carcinoma.

## Conclusions

In conclusion, our comprehensive pan-cancer analysis of ESRG across various cancer types demonstrated its potential to be used as a diagnostic biomarker in COAD, LUSC, READ, and UCEC and a promising prognostic biomarker in LGG. Furthermore, our findings figured out relatively significant variations in ESRG expression between normal and different cancer stages in COAD, LUSC, and READ. However, in UCEC, ESRG was significantly expressed only in stage 1. Moreover, our results demonstrate a significant negative correlation between ESRG expression and the abundance of CD8+ T cells in COAD, READ, LUSC, and UCEC. Additionally, ESRG was mutated in 0.77 (<1%) of the queried samples, and the most prevalent ESRG mutations are deep deletion mutations, followed by amplification.
